# Prevalence of Hearing Loss and Hearing Aid Use Among Persons Living With Dementia in the US

**DOI:** 10.1001/jamanetworkopen.2024.40400

**Published:** 2024-10-21

**Authors:** Carrie L. Nieman, Emmanuel E. Garcia Morales, Alison R. Huang, Nicholas S. Reed, Sevil Yasar, Esther S. Oh

**Affiliations:** 1 Department of Otolaryngology–Head and Neck Surgery, Johns Hopkins University School of Medicine, Baltimore, Maryland; 2 John Hopkins Cochlear Center for Hearing and Public Health, Johns Hopkins Bloomberg School of Public Health, Baltimore, Maryland; 3 Johns Hopkins University School of Nursing, Baltimore, Maryland; 4 Department of Epidemiology, Johns Hopkins Bloomberg School of Public Health, Baltimore, Maryland; 5 Division of Geriatric Medicine and Gerontology, Department of Medicine, Johns Hopkins University School of Medicine, Baltimore, Maryland; 6 Department of Psychiatry and Behavioral Sciences, Johns Hopkins University School of Medicine, Baltimore, Maryland; 7 Division of Neuropathology, Department of Pathology, Johns Hopkins University School of Medicine, Baltimore, Maryland

## Abstract

**Importance:**

Dementia and hearing loss are prevalent and represent public health priorities, but little is known about the prevalence of hearing loss among persons living with dementia at a population level.

**Objective:**

To estimate the prevalence of hearing loss and hearing aid use among a nationally representative sample of persons living with dementia.

**Design, Setting, and Participants:**

In this cross-sectional study, we analyzed data from Medicare beneficiaries who participated in round 11 of the National Health and Aging Trends Study (NHATS). The analytic sample included participants who had complete audiometric data and a dementia classification, and survey weights were applied to yield nationally representative estimates. Data were collected from June to November 2021, and analyzed from October 12, 2023, to February 27, 2024.

**Exposures:**

Audiometric measures of hearing loss and self-reported hearing aid use.

**Main Outcomes and Measures:**

National prevalence estimates of possible and probable dementia and hearing loss were estimated by applying NHATS survey weights of sex and age to the population aged 71 years or older based on the US Census Bureau’s 2020 American Community Survey data.

**Results:**

The analytic sample comprised 2613 NHATS participants (74.6% aged 71-79 years and 25.4% aged ≥80 years; 52.9% female). Overall prevalence of hearing loss among participants with dementia was estimated at 79.4% (95% CI, 72.1%-85.3%). The prevalence of hearing loss increased with age, from 61.1% (95% CI, 37.7%-80.2%) among participants aged 71 to 74 years to 94.2% (95% CI, 88.8%-97.0%) among those aged 85 years or older. Among participants with hearing loss, 21.7% (95% CI, 16.2%-28.3%) reported hearing aid use.

**Conclusions and Relevance:**

In this cross-sectional study, the findings suggest that hearing loss is highly prevalent among individuals with dementia, with a low prevalence of hearing aid use. These findings reveal a potential opportunity for intervention.

## Key Points

**Question:**

What is the prevalence of hearing loss and hearing aid use among older adults living with dementia in the US?

**Findings:**

In this cross-sectional analysis of 2613 participants aged 71 years or older from round 11 of the National Health and Aging Trends Study, a weighted 79.4% with dementia had clinically significant hearing loss, increasing with age to 94.2% in participants 85 years or older. Overall, 21.7% of participants with hearing loss reported using hearing aids.

**Meaning:**

These findings indicate a high prevalence of audiometric hearing loss yet limited use of hearing aids among persons living with dementia, suggesting an opportunity for intervention.

## Teaser

This cross-sectional study estimates the prevalence of hearing loss and hearing aid use among a nationally representative sample of older adults living with dementia in the US.

## Introduction

Dementia represents a significant and growing public health challenge due to an aging global population. The number of individuals with dementia is expected to increase from 57.4 million in 2019 to 152.8 million in 2050.^1,2^ As another age-related condition, hearing loss represents one of the largest potentially modifiable risk factors for dementia at a population level.^1^ Hearing loss is highly prevalent, increases with age, and has been repeatedly and independently associated with dementia and accelerated cognitive decline.^1,3,4^ Recent evidence has indicated the potential for hearing care intervention to slow cognitive decline among individuals most at risk.^5^ Among persons living with dementia (PLWD), sensory impairments, such as age-related hearing loss, are common and have been independently associated with an increased risk of neuropsychiatric symptoms and functional decline.^6,7,8,9^ However, nationally representative estimates of audiometric hearing loss prevalence among PLWD are lacking.^9^ As both dementia and hearing loss have been identified as public health priorities that demand a public health–driven approach,^1,10,11^ a rigorous understanding of the prevalence and population estimates of persons living with concurrent dementia and hearing loss are needed.

Relatively little is known about the prevalence of hearing loss among PLWD at a population level. Current understanding of hearing loss among PLWD is often based on convenience samples primarily from specialized memory clinics.^6,12^ Furthermore, hearing status is frequently measured by self- or proxy-reported measures, which often underestimate the presence and severity of audiometric hearing loss.^13^ Audiometric evaluations, including pure-tone audiometry on tablet-based platforms, are feasible and accurate for most PLWD.^14^ Among population-based studies, challenges include poor dementia adjudication, limited audiometric evaluation of hearing, limited sampling of the oldest patients (aged ≥85 years; a population that often has the highest prevalence of both dementia and hearing loss), and barriers to study participation among these populations.

As hearing loss is increasingly recognized as a potentially modifiable risk factor for cognitive decline and incident dementia, hearing care represents a potential form of primary prevention.^1,5^ However, we must also consider the role of hearing in the tertiary prevention of the sequela of dementia. To understand and address hearing health among PLWD from a public health perspective, we aimed to estimate the prevalence of hearing loss among PLWD in a nationally representative, community-dwelling sample using audiometric hearing measures and to characterize the potential unmet hearing health needs of PLWD.

## Methods

### Study Participants

In this cross-sectional study, we used data from round 11 of the National Health and Aging Trends Study (NHATS) completed in 2021. The NHATS protocol was approved by the Johns Hopkins University Institutional Review Board, and written informed consent was obtained from all study participants. The current study was considered an analysis of secondary, deidentified, publicly available data; thus, institutional review board approval was not required in accordance with the Common Rule. The study followed the Strengthening the Reporting of Observational Studies in Epidemiology (STROBE) reporting guideline.^15^

The NHATS is a nationally representative longitudinal study of older Medicare beneficiaries. Medicare is a federally funded program that provides health insurance for individuals aged 65 years or older and for some younger people with certain health conditions. In 2022, 98.9% of older adults in the US were covered by Medicare.^16^

Starting in 2011, the NHATS interviewed participants annually. The study sample was replenished in 2015 to maintain national representation. As a result, by the time of round 11 in 2021, the youngest participant in the sample was aged 71 years. Defining characteristics of NHATS include in-home visits that decrease barriers to ongoing inclusion, a longitudinal design, and oversampling of adults who may traditionally be excluded from population-based studies, such as the oldest beneficiaries.

A total of 3466 community-dwelling and institutionalized (eg, living in skilled nurse facilities, living in nursing homes) older adults (aged ≥71 years) were interviewed from June to November 2021. To estimate the prevalence and number of older adults with dementia, our analytic sample first excluded participants with missing dementia classification (n = 78) and those with incomplete 4-frequency audiometric data for both ears (n = 775), yielding an initial analytic sample of 2613 (Figure). To estimate the proportion of PLWD who also had hearing loss, we restricted our sample to older adults who were identified as having dementia, resulting in a final analytic sample of 394 participants to estimate the prevalence of hearing loss among this group. An additional 29 participants had missing information regarding race and ethnicity; these participants were excluded only from our prevalence estimates regarding race and ethnicity but were included in all other prevalence estimates.

**Figure.**
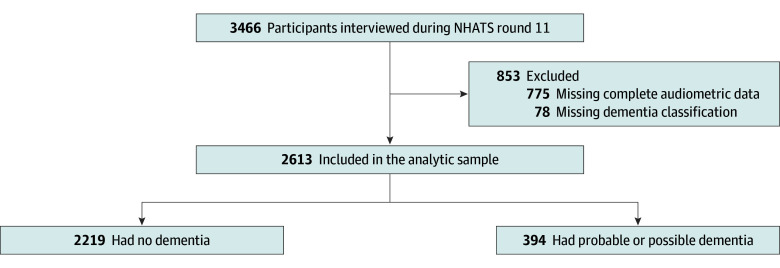
Flow Diagram for Analytic Sample Definition NHATS indicates National Health and Aging Trends Study.

### Hearing-Related Measures

As part of the round 11 follow-up, participants were offered pure-tone air conduction audiometric testing using a tablet-based portable audiometer (SHOEBOX Ltd). We computed the 4-frequency (0.5, 1, 2, and 4 kHz) pure-tone average (PTA) for the better hearing ear. We then classified participants into 4 categories: no hearing loss (PTA, ≤25 dB hearing level [dB HL]), mild hearing loss (PTA, >25 to ≤40 dB HL), moderate hearing loss (PTA, >40 to ≤60 dB HL), and severe or worse hearing loss (PTA, >60 dB HL) per former World Health Organization (WHO) standards.^17^ For sensitivity analyses, we categorized participants according to the most recent WHO guidelines: no hearing loss (PTA, <20 dB HL), mild hearing loss (PTA, ≥20 to <35 dB HL), moderate hearing loss (PTA, ≥35 to <50 dB HL), moderately severe hearing loss (PTA, ≥50 to <65 dB HL), and severe or greater hearing loss (PTA, ≥65 dB HL).^18,19^ Hearing aid use was assessed based on the participant’s answer to the question, “In the last month, have you used a hearing aid or other hearing device? (yes, no).”

### Dementia Status

Dementia status was empirically defined by NHATS based on proxy report of dementia; the Eight-Item Informant Interview to Differentiate Aging and Dementia (AD8)^20^; and cognitive test scores in the domains of memory, orientation, and executive function.^21^ Participants were encouraged to use their hearing aids during the interview if they ordinarily used them, and interviewers noted difficulties in administration of the cognition section related to trouble hearing in 29 participants. Participants with (1) self-reported or a proxy-reported diagnosis of dementia, (2) an AD8 score of at least 2, or (3) cognitive test scores 1.5 SDs or less below the mean in at least 2 of the 3 cognitive domains (memory [immediate and delayed 10-word recall], orientation [correctly stating the date in month, year, and day of the week and naming the US president and vice president], and executive function [clock drawing test]) were classified as having probable dementia.

Participants with an AD8 score less than 2 or a lack of a dementia diagnosis but a cognitive test score 1.5 SD or less below the mean in 1 of the 3 cognitive domains were classified as having possible dementia. Participants not meeting any of these criteria were classified as having no dementia. The diagnoses for probable and possible dementia have been developed and validated by NHATS, demonstrating a sensitivity and specificity of 65.7% and 87.2%, respectively, for probable dementia and 85.7% and 61.6%, respectively, for combined probable and possible dementia.^21^ For our main analyses, we combined participants with probable and possible dementia in a single dementia group.

All questionnaires, including all cognitive tests, were translated into Spanish and administered as such to Spanish-speaking respondents with limited English proficiency. Only 9 participants identified as having probable or possible dementia were administered the translated questionnaire.

### Statistical Analysis

The data analysis was performed from October 12, 2023, to February 27, 2024. We examined differences in participant characteristics by inclusion and exclusion in the analytic sample and among those in the analytic sample by dementia status. Then, we estimated the prevalence of dementia (possible and probable) among older Medicare beneficiaries by age group (71-74, 75-79, 80-84, and ≥85 years) and self-reported sex (female, male) and race and ethnicity (Hispanic, non-Hispanic Black, and non-Hispanic White; estimations for participants with other races and ethnicities were omitted from prevalence analyses due to cell size smaller than 10). Race and ethnicity data were analyzed because racial and ethnic minority individuals have been found to experience disproportionate dementia-associated burden.^22^ Finally, among individuals with dementia, we estimated the prevalence of hearing loss severity by age, sex, and race and ethnicity. In sensitivity analyses, we estimated the prevalence of hearing loss only among participants by probable (the stricter of the 2 dementia definitions) and possible dementia status and among those with possible and probable dementia using the most recent WHO guidelines for hearing loss.

All analyses included round 11 survey weights to account for the NHATS complex survey design. To obtain national prevalence estimates, survey weights were sex and age standardized to the population aged 71 years or older living in the continental US based on the US Census Bureau 2020 American Community Survey data, as per NHATS guidelines.^23^ Population totals were calculated by extrapolating the estimated prevalence of dementia among NHATS participants to the number of individuals identified in the census data. Data were analyzed using Stata, version 17 (StataCorp LLC).

## Results

In the sample of 2613 Medicare beneficiaries aged 71 years or older with available dementia and audiometric data, a weighted 52.9% of the analytic sample self-identified as female (vs 47.1% male), and 7.2% self-identified as Black, 6.7% as Hispanic, 82.6% as White, and 3.5% as other race or ethnicity (including American Indian, Alaska Native, Asian, Native Hawaiian, Pacific Islander, and multiple races or ethnicity groups). Weighted estimates revealed that compared with participants with no dementia, those with probable and possible dementia were older (aged ≥85 years, 30.1% [95% CI, 24.4%-35.9%] vs 9.7% [95% CI, 8.7%-10.7%]), more likely to be female (60.2% [95% CI, 51.3%-69.2%] vs 52.2% [95% CI, 49.3%-55.1%]), and less likely to self-identify as White (66.0% [95% CI, 58.1%-73.9%] vs 84.4% [95% CI, 81.4%-87.4%]) (Table 1). Participants excluded from our analytic sample were older and more likely to be female (eTable 1 in Supplement 1). An analysis of attrition between rounds 10 and 11 of NHATS showed that a higher proportion of participants identified as having probable or possible dementia died during the study rounds (21.2% vs 4.0% without dementia) or were lost to follow-up (10.9% vs 4.2% without dementia) (eTable 3 in Supplement 1).

**Table 1.** Participant Characteristics by Dementia Status (N = 2613), Round 11 of the National Health and Aging Trends Study

Characteristic	Weighted % (95% CI)		
	Total	No dementia	Probable and possible dementia
Age group, y			
71-74	38.5 (35.7-41.5)	40.5 (37.4-43.6)	20.5 (12.2-28.9)
75-79	36.1 (33.2-39.1)	36.7 (33.6-39.7)	30.8 (23.2-38.4)
80-84	13.7 (12.6-14.9)	13.2 (11.9-14.4)	18.5 (14.9-22.1)
≥85	11.7 (10.7-12.7)	9.7 (8.7-10.7)	30.1 (24.4-35.9)
Sex			
Female	52.9 (50.2-55.7)	52.2 (49.3-55.1)	60.2 (51.3-69.2)
Male	47.1 (44.3-49.8)	47.8 (44.9-50.7)	39.8 (30.8-48.7)
Race and ethnicity^a^			
Black	7.2 (6.0-8.5)	6.6 (5.4-7.8)	12.5 (8.8-16.3)
Hispanic	6.7 (4.7-9.4)	5.5 (3.3-7.7)	17.9 (11.1-24.7)
White	82.6 (79.3-85.5)	84.4 (81.4-87.4)	66.0 (58.1-73.9)
Other^b^	3.5 (2.4-5.2)	3.5 (2.3-5.3)	NA^c^

Abbreviation: NA, not applicable.

^a^ A total of 29 participants had missing information about race or ethnicity (no dementia group, 26 participants; probable and possible dementia group, 3 participants).

^b^ Other includes American Indian, Alaska Native, Asian, Native Hawaiian, Pacific Islander, and multiple races or ethnicity groups.

^c^ Estimates omitted due to cell size smaller than 10 observations.

Additionally, we estimated an overall prevalence of dementia of 9.7% (95% CI, 8.2%-11.5%), corresponding to 3.2 million (95% CI, 2.7-3.8 million) older adults (Table 2). Our results show that prevalence of dementia increased with age, as only 5.2% (95% CI, 2.5%-7.9%) of adults aged 71 to 74 years but 25.1% (95% CI, 22.0%-28.2%) of those aged 85 years or older were identified as having dementia. We found that the prevalence of dementia was greater among women (11.1%; 95% CI, 8.6%-13.5%) than men (8.2%; 95% CI, 6.0%-10.4%) and greater among Black (16.9%; 95% CI, 12.6%-22.1%) and Hispanic (25.8%; 95% CI, 17.4%-36.6%) older adults than among their White counterparts (7.7%; 95% CI, 6.3%-9.4%).

**Table 2.** Prevalence and Population-Weighted Estimates of Hearing Status Among Persons Living With Dementia in the US, Round 11 of the National Health and Aging Trends Study

Variable	Prevalence of dementia among the current sample population (95% CI)		Prevalence of hearing loss among older adults with probable and possible dementia by hearing loss severity (95% CI)					
	Probable and possible dementia, %	Millions of people	Any hearing loss (PTA, >25 dB HL), %	Millions of people	Normal hearing (PTA, ≤25 dB HL), %	Mild (PTA, >25 to ≤40 dB HL), %	Moderate or worse (PTA, >40 dB HL), %
Overall	9.7 (8.2-11.5)	3.2 (2.7-3.8)	79.4 (72.1-85.3)	2.6 (2.1-3.1)	20.5 (14.7- 27.9)	32.1 (25.2-39.9)	47.4 (40.8-54.0)
Age group, y							
71-74	5.2 (2.5-7.9)	0.6 (0.3-0. 9)	61.1 (37.7-80.2)	0.4 (0.1-0.6)	38.9 (16.3-61.5)	NA^a^	NA^a^
75-79	8.3 (5.9-10.7)	0.8 (0.6-1.0)	73.5 (57.8-84.9)	0.6 (0.4-0.8)	26.5 (12.7-40.3)	25.9 (13.0-38.9)	47.6 (31.2-64.0)
80-84	13.2 (10.2-16.2)	0.8 (0.6-1.0)	85.8 (75.7-92.1)	0.7 (0.5-0.9)	14.2 (6.1-22.2)	46.2 (31.5-60.9)	39.6 (27.3-52.0)
≥85	25.1 (22.0-28.2)	1.5 (1.3-1.7)	94.2 (88.8-97.0)	1.4 (1.2-1.6)	5.8 (3.0-11.1)	28.2 (20.9-36.9)	66.0 (56.7-74.1)
Sex							
Female	11.1 (8.6-13.5)	2.1 (1.6-2.5)	74.3 (63.5-82.8)	1.5 (1.1-1.9)	25.7 (16.0-35.4)	38.5 (27.9-49.0)	35.8 (28.2-43.4)
Male	8.2 (6.0-10.4)	1.2 (0.9-1.5)	87.2 (77.6-93.1)	1.0 (0.7-1.3)	12.7 (5.2-20.3)	22.4 (14.6-30.2)	64.9 (56.5-73.2)
Race and ethnicity^b^							
Black	16.9 (12.6-22.1)	0.4 (0.3-0.5)	51.7 (39.1-64.1)	0.2 (0.1-0.3)	48.3 (35.5-61.0)	32.6 (20.7-44.5)	19.1 (12.2-26.0)
Hispanic	25.8 (17.4-36.6)	0.6 (0.4-0.8)	76.8 (47.9-92.3)	0.4 (0.2-0.7)	NA^a^	23.5 (7.4-39.5)	53.4 (30.8-75.9)
White	7.7 (6.3-9.4)	2.1 (1.7-2.5)	85.1 (76.8-90.7)	1.8 (1.4-2.2)	14.9 (8.0-21.8)	33.7 (25.5-41.9)	51.4 (43.4-59.3)

Abbreviations: dB HL, dB hearing level; NA, not available; PTA, pure-tone average.

^a^ Estimates omitted due to cell size smaller than 10 observations.

^b^ A total of 3 participants in the probable and possible dementia group had missing information about race and ethnicity (1 in the mild hearing loss group and 2 in the moderate or worse hearing loss group).

We estimated that 79.4% (95% CI, 72.1%-85.3%) of older adults with dementia experienced some degree of audiometric hearing loss (mild, 32.1% [95% CI, 25.2%-39.9%]; moderate or worse, 47.4% [95% CI, 40.8%-54.0%]) (Table 2). Prevalence and severity of hearing loss among participants with dementia increased with age, with 61.1% (95% CI, 37.7%-80.2%) of those aged 71 to 74 years and 94.2% (95% CI, 88.8%-97.0%) of those aged 85 years or older having some degree of hearing loss. In addition, 47.6% (95% CI, 31.2%-64.0%) of those aged 75 to 79 years and 66.0% (95% CI, 56.7%-74.1%) of those aged 85 years or older experienced moderate or worse hearing loss. Additionally, the prevalence of hearing loss was not only greater among men (87.2%; 95% CI, 77.6%-93.1%) compared with women (74.3%; 95% CI, 63.5%-82.8%) but also higher among men (64.9%; 95% CI, 56.5%-73.2%) experiencing more severe (moderate or worse) hearing loss compared with women experiencing more severe hearing loss (35.8%; 95% CI, 28.2%-43.4%).

Among older adults with dementia, the prevalence of hearing loss was greater among White (85.1%; 95% CI, 76.8%-90.7%) and Hispanic (76.8%; 95% CI, 47.9%-92.3%) participants compared with Black participants (51.7%; 95% CI, 39.1%-64.1%). In terms of severity, while a similar proportion of White (51.4%; 95% CI, 43.4%-59.3%) and Hispanic (53.4%; 95% CI, 30.8%-75.9%) older adults had moderate or worse hearing loss, fewer Hispanic participants (23.5%; 95% CI, 7.4%-39.5%) experienced a mild to moderate degree of hearing loss compared with White participants (33.7%; 95% CI, 25.5%-41.9%).

In sensitivity analyses, we found that the overall prevalence of hearing loss among participants with probable dementia (the stricter definition) was 74.2% (95% CI, 62.1%-83.5%) and among those with possible dementia, 84.6% (95% CI, 74.9%-91.1%). Our findings with respect to higher prevalence of hearing loss among older age groups and among White individuals were robust to different definitions of hearing loss (eTable 2 in Supplement 1).

Among participants with audiometric hearing loss and dementia, 21.7% (95% CI, 16.2%-28.3%) reported hearing aid use. We were unable to complete further analyses related to hearing aid use by demographic factors, such as race and ethnicity, due to limitations in sample size.

## Discussion

In this cross-sectional study of a nationally representative cohort of Medicare beneficiaries with complete audiometric data and a classification of dementia, we found that the prevalence of clinically significant hearing loss was high among PLWD (79.4%), with the highest prevalence among the oldest individuals (94.2% among individuals aged ≥85 years). The prevalence of hearing loss among our participants with dementia is comparable with nationally representative estimates of older adults by age group.^3^ Prior studies of PLWD estimated the prevalence of hearing loss from 60% to more than 90% based on audiometric testing of patients within tertiary memory clinics.^6,12^ Prior estimates of hearing loss prevalence that relied on proxy-rated hearing status among individuals with cognitive impairment have been as low as 14%, where care partners and health care clinicians often underestimate the presence and severity of hearing loss among PLWD.^13,24,25,26,27^

While we found audiometric hearing loss to be highly prevalent among PLWD, hearing aid use was low at 21.7%. Compared with estimates of hearing aid use among the broader population of older adults, the prevalence of hearing aid use among PLWD was lower compared with findings from NHATS (29.2%) but comparable with findings from the National Health and Nutrition Examination Survey (19.3%), although both estimates may represent a younger population.^3,28,29^ Among NHATS participants with audiometric hearing loss aged 80 to 85 years, hearing aid use was estimated to be 31.2% compared with 36.9% among individuals aged 85 years or older.^3^

### Limitations

This study has several limitations. First, barriers exist to hearing care, including among PLWD, and overall disparities in care exist by race, ethnicity, and socioeconomic position.^30,31,32^ Limitations associated with sample size precluded further analysis to understand the prevalence of hearing aid use among PLWD who identified as a racial or ethnic minority, communities that experience disproportionate dementia-related burden.^22^ Second, the design of NHATS allows for nationally representative estimates, but the underlying sample is small (394 participants) and may limit the generalizability of the findings. Third, our prevalence estimates with respect to the Hispanic population should be interpreted with caution as the NHATS design did not oversample for this population. Fourth, the classification of dementia status used within NHATS is limited and relies in part on participants’ ability to hear a word list. While few participants (n = 29) were noted to have difficulty hearing the word list presented by the interviewer, the noted difficulty may have influenced the categorization of some participants’ cognitive status. Fifth, the classification of dementia status is not based on a robust neuropsychological evaluation, and diagnoses remained possible and probable. However, given the sensitivity and specificity of the NHATS dementia measures, this study’s estimates may represent conservative estimates of a population typically not included in population-based studies. Given the longitudinal nature of NHATS as an exploratory exercise, we analyzed attrition numbers between round 10 and round 11 by round 10’s dementia status. We found that a higher proportion of participants identified as having probable and possible dementia died during the study rounds or were lost to follow-up, further reinforcing our suspicions that our calculations may be conservative. Finally, we found that the prevalence of hearing loss was lower among participants with probable dementia than among those with possible dementia. Thus, by including participants with possible dementia, our prevalence results for PLWD may be overestimated.

## Conclusions

In this cross-sectional study of a nationally representative sample of older adults in the US, our findings suggest that hearing loss is highly prevalent among PLWD yet frequently unaddressed. Hearing loss may represent one of the most common comorbidities of dementia and must be included in consideration of the health and well-being of PLWD. With a growing understanding of the importance of sensory health within aging and cognitive health, the unmet hearing care needs of persons living with concurrent dementia and hearing loss is a public health priority. Future research is needed to aid in the identification and management of hearing loss among PLWD through clinical and public health–driven approaches.

## Notes

Supplement 1. **eTable 1.** Characteristics of Study Nondeceased Participants in Round 11 of the National Health and Aging Trends Study by Inclusion and Exclusion Status in the Initial Analytic Sample **eTable 2.** Prevalence of Hearing Loss Among Participants With Dementia by Hearing Loss Severity According to Current WHO Standards **eTable 3.** Participants With Available Dementia Information at Round 10 by Study Participation Status and Residential Status in Round 11 of the National Health and Aging Trends Study (NHATS)

Supplement 2.
Data Sharing Statement
