# Supplementary material for: Prevalence of Hearing Loss and Hearing Aid Use Among Persons Living With Dementia in the US
Source: JAMA Netw Open. 2024 Oct 21;7(10):e2440400. doi: 10.1001/jamanetworkopen.2024.40400 (PMC11581568; doi:10.1001/jamanetworkopen.2024.40400)
Supplement: Supplement 1. — eTable 1. Characteristics of Study Nondeceased Participants in Round 11 of the National Health and Aging Trends Study by Inclusion and Exclusion Status in the Initial Analytic Sample eTable 2. Prevalence of Hearing Loss Among Participants With Dementia by Hearing Loss Severity According to Current WHO Standards eTable 3. Participants With Available Dementia Information at Round 10 by Study Participation Status and Residential Status in Round 11 of the National Health and Aging Trends Study (NHATS) [file jamanetwopen-e2440400-s001.pdf]

Supplemental Online Content

Nieman CL, Garcia Morales EE, Huang A, Reed NS, Yasar S, Oh ES. Prevalence of hearing loss and hearing aid use among persons living with dementia in the US. *JAMA Netw Open*. 2024;7(10):e2440400. doi:10.1001/jamanetworkopen.2024.40400

eTable 1. Characteristics of Study Nondeceased Participants in Round 11 of the National Health and Aging Trends Study by Inclusion and Exclusion Status in the Initial Analytic Sample

eTable 2. Prevalence of Hearing Loss Among Participants With Dementia by Hearing Loss Severity According to Current WHO Standards

eTable 3. Participants With Available Dementia Information at Round 10 by Study Participation Status and Residential Status in Round 11 of the National Health and Aging Trends Study (NHATS)

This supplemental material has been provided by the authors to give readers additional information about their work.

© 2024 Nieman CL et al. *JAMA Network Open.*

eTable 1. Characteristics of Study Nondeceased Participants in Round 11 of the National Health and Aging Trends Study by Inclusion and Exclusion Status in the Initial Analytic Sample

	Total Sample	Excluded Participants	Included Participants
	N=3,466	N=853	N=2,613
**Age**			
71 – 74	12.8% (445)	8.6% (73)	14.2% (372)
75 – 79	28.5% (987)	22.6% (193)	30.4% (794)
80 – 84	24.4% (847)	21.6% (184)	25.4% (663)
85 +	34.2% (1,187)	47.2% (403)	30.0% (784)
**Sex**			
Male	41.2% (1,428)	34.0% (290)	43.6% (1,138)
Female	58.8% (2,038)	66.0% (563)	56.4% (1,475)
**Race/Ethnicity** a\			
African American	20.4% (697)	26.6% (222)	18.4% (475)
Hispanic	4.9% (168)	6.5% (54)	4.4% (114)
White	72.2% (2,469)	64.0% (535)	74.8% (1,934)

a\ A total of N=46 participants had missing information about race or ethnicity. N=17 from the excluded group and N=29 from the included group.

© 2024 Nieman CL et al. *JAMA Network Open.*

eTable 2. Prevalence of Hearing Loss Among Participants With Dementia by Hearing Loss Severity According to Current WHO Standards [17]

		Hearing Loss Severity			
	Normal Hearing PTA <20	Mild 20 <= PTA < 35	Moderate 35<=PTA < 50	Moderately Severe or More 50<=PTA	Any Hearing Loss PTA=>20
**Overall**	8.4(5.2,13.4)	33.1 (26.0,41.1)	30.9 (24.0,38.8)	27.6 (20.7,35.8)	91.6 (86.6,94.8)
**Age**					
71 – 74	b\	b\	b\	b\	78.4 (61.7,95.1)
75 – 79	8.8 (1.4,16.2)	33.7 (20.2,47.3)	28.7 (14.4,42.9)	28.8 (12.7,44.9)	91.2 (83.8,98.6)
80 – 84	b\	35.3 (23.6,47)	37.2 (25.5,48.9)	21.4 (13.1,29.8)	93.9 (89.3,98.4)
85 +	b\	23.7 (16.8,32.4)	34.4 (26.2,43.6)	41.4 (32.9,50.5)	99.5 (97.6,100)
**Sex**					
Male	7.5 (2.5,12.4)	21.6 (14.6,28.6)	29.5 (19.3,39.7)	41.4 (30.4,52.4)	92.5 (87.6,97.5)
Female	9.1 (3,15.1)	40.7 (30.1,51.3)	31.8 (22.7,41)	18.4 (11.5,25.4)	90.9 (84.9,97)
**Race/Ethnicity** a\					
African American	27.4 (13.9,40.8)	38.2 (25.8,50.7)	26.1 (17.6,34.5)	8.3 (3.2,13.4)	72.6 (59.2,86.1)
Hispanic	b\	b\	46.8 (27.9,65.7)	21.5 (5.6,37.4)	96 (88.2,100.0)
White	6.9 (1.7,12.2)	31.2 (22.1,40.3)	29.3 (20.4,38.1)	32.6 (24.1,41.1)	93.1 (87.8,98.3)

a\ A total of N=3 participants had missing information about race or ethnicity. N=1 from the mild hearing loss group, N=2 from the moderately severe or more hearing loss group.

b\ Estimates omitted due to cell size smaller than 10 observations.

© 2024 Nieman CL et al. *JAMA Network Open.*

eTable 3. Participants With Available Dementia Information at Round 10 by Study Participation Status and Residential Status in Round 11 of the National Health and Aging Trends Study (NHATS)

	Participation and Residential Status during Round 11			
Dementia Status Round 10	Community Dwelling	Residential Facility	Deceased	Lost to Follow-Up
Probable and Possible dementia	488 (52.1%)	148 (15.8%)	198 (21.2%)	62 (10.9%)
No dementia	2,597 (85.8%)	175 (5.7%)	123 (4.0%)	130 (4.2%)

© 2024 Nieman CL et al. *JAMA Network Open.*
