# Supplementary material for: Prevalence of Hearing Loss and Hearing Aid Use Among Persons Living With Dementia in the US
Source: JAMA Netw Open. 2024 Oct 21;7(10):e2440400. doi: 10.1001/jamanetworkopen.2024.40400 (PMC11581568; doi:10.1001/jamanetworkopen.2024.40400)
Supplement: Supplement 2. — Data Sharing Statement [file jamanetwopen-e2440400-s002.pdf]

Data Sharing Statement

Nieman. Prevalence of Hearing Loss and Hearing Aid Use Among Persons Living With Dementia in the US. *JAMA Netw Open*. Published October 21, 2024.
doi:10.1001/jamanetworkopen.2024.40400

Data
Data available: No

Additional Information
Explanation for why data not available: Data are available publicly.
